# Associations of Lower-Limb Phase Angle with Locomotion and Motor Function in Japanese Community-Dwelling Older Adults

**DOI:** 10.3390/geriatrics8060121

**Published:** 2023-12-14

**Authors:** Daisuke Homma, Izumi Minato, Norio Imai, Dai Miyasaka, Yoji Horigome, Hayato Suzuki, Yoichiro Dohmae, Naoto Endo

**Affiliations:** 1 Division of Orthopaedic Surgery, Niigata University Graduate School of Medical and Dental Sciences, Niigata 951-8510, Japan; 2 Department of Rehabilitation, Niigata Bandai Hospital, Niigata 950-0909, Japan; 3 Division of Orthopaedic Surgery, Niigata Rinko Hospital, Niigata 950-0051, Japan; 4 Division of Comprehensive Musculoskeletal Medicine, Niigata University Graduate School of Medical and Dental Sciences, Niigata 951-8510, Japan; 5 Division of Orthopaedic Surgery, Niigata Bandai Hospital, Niigata 950-0909, Japan; 6 Division of Orthopaedic Surgery, Niigata Prefectural Tsubame Rosai Hospital, Tsubame, Niigata 959-1228, Japan

**Keywords:** Kihon Checklist, 25-Geriatric Locomotive Function Scale, locomotive syndrome, healthy life expectancy, frailty

## Abstract

Whole-body phase angle (PhA) is associated with motor function and geriatric diseases. However, it was unclear which parts of the upper- or lower-limb PhA were involved. This study investigated the differences in the PhA of the upper and lower extremities and their relationships with frailty, locomotive syndrome indices, and motor function in community-dwelling older adult participants. This study was a cross-sectional observational study. In 69 community-dwelling older adults, the PhA at each measurement site (whole body, upper limbs, and lower limbs), motor function, Kihon Checklist (KCL), and 25-Geriatric Locomotive Function Scale (GLFS-25) were measured. This study examined differences in each PhA and its relationship with motor function and geriatric diseases. Multiple regression analysis was performed using GLFS-25 as the dependent variable and sex and lower-limb PhA as independent variables. In this cohort (mean age: 72.3 ± 5.7 years; 18 males and 51 females), lower-limb PhA was significantly lower than upper-limb PhA. Unlike other PhAs, lower-limb PhA was related to grip strength, TUG, F/w, and GLFS-25. Multiple regression analysis showed that the lower-limb PhA was independently related to GLFS-25. Cellular health of the lower extremity is associated with gait, standing function, and indicators of locomotive syndrome.

## 1. Introduction

The global population is increasing, and this growth is characterized by an increase in the aging population as well as a decline in the fertility rate [1]. Japan has the world’s largest proportion of the aging population [2]; the proportion of people aged ≥65 years reached 9.3% in 2020, which constitutes a significant increase from 6.9% in 2000 and 5.1% in 1950. Furthermore, the percentage of the world’s older adult population is projected to reach 15.9% and 22.4% by 2050 and 2100, respectively [1]. The increase in the older population (aged ≥ 65 years) is expected to place a significant burden on the healthcare system because of difficulties in self-care, independent living, and walking [3]. Therefore, urgent measures are needed to address global population aging and extend healthy life expectancy, which is a measure of the length of time a person can live independently [3]. To extend healthy life expectancy, it is necessary to implement measures against age-related diseases, such as frailty and locomotive syndrome (LS). Frailty is defined as an intermediate state of reduced homeostasis to stress (a state between robust health and the need for nursing care) [4], and LS is defined as a state of reduced mobility and an increased risk of disability due to disorders of the locomotor system [5,6]. Frailty and LS are important diseases that need to be prevented in Japan; they should be assessed at an early stage for timely prevention or management. Both conditions involve musculoskeletal organs and muscle degeneration, such as sarcopenia; therefore, objective assessment of skeletal muscles is necessary.

A simple and noninvasive method for evaluating muscles in a clinical setting is the multi-frequency bioelectrical impedance analysis (BIA) method [7]. Conventional methods for objectively evaluating muscles include magnetic resonance imaging [8], computed tomography [9], and muscle biopsy [10]; however, invasive procedures, large equipment, long time restraints, and the need for skilled technicians have made it difficult to perform measurements in the clinical setting [11]. Echo intensity obtained from ultrasound images has also been used for muscle evaluation as a noninvasive and simple method [12], although concerns exist regarding the reproducibility of the results because each examiner has a different measurement technique [11]. In contrast, the BIA method involves an instrument that can evaluate muscle volume and quality from weak electrical resistance in vivo and is becoming a popular evaluation method [13]. The phase angle (PhA), which can be measured using the BIA method, reflects the physiological function of cells and is attracting attention [14]. The PhA reflects the entire tissue volume, including muscle and water. As the PhA is related to muscle strength and physical function [15,16], individuals with high PhA values are considered to have high muscle density and good muscle quality, which may indicate good motor function.

Previous literature has reported several studies that used whole-body PhA as an indicator of whole-body cellular health, examining the effects of training and investigating its relationship to the occurrence of disability in older adults [17,18]. Furthermore, Tanaka et al. investigated the relationship between PhA, frailty, and locomotive syndrome in older adults in 2019, suggesting that locomotive syndrome and frailty are related to PhA [19]. These results suggest that systemic PhA is related to the occurrence of disabilities, motor function, and geriatric diseases in older adults. However, PhA is based on values for the whole body, and it was not clear whether the upper or lower limbs were associated with greater motor function or with indicators of geriatric diseases. We believe that by using PhA as an indicator to elucidate this point, we can obtain effective knowledge for prevention and improvement by focusing on the health of cells in the upper or lower limbs. In fact, we previously measured PhA at different measurement sites to compare prefrail and strong people and found that PhA in the lower limbs was related to balance and walking ability [20]. However, our previous studies did not establish a difference in PhA values between the upper and lower limbs, and we were also unable to investigate the relationship with indicators of geriatric diseases. Based on these results, we hypothesized that PhA differs depending on the measurement site, such as the upper or lower limbs, and that related indicators of geriatric diseases and motor function also differ. Moreover, questionnaires are used to evaluate geriatric diseases, such as frailty and LS; however, because most questionnaires evaluate mobility, such as gait function and activity, PhA may be related to the lower limbs rather than the upper limbs. By clarifying these aspects, we believe it is possible to propose the importance of site-specific measurements when evaluating PhA and develop an effective evaluation index for the objective evaluation of muscles related to geriatric diseases.

This study aimed to investigate the differences in the PhA of the upper and lower limbs and their relationships with frailty, LS indices, and motor function in community-dwelling older adult participants. The insights from this study will enable the consideration of intervention points in terms of muscle quality and clarify the relationship between frailty and LS and PhA, which can be indicators of muscle quality and thus contribute to the extension of healthy life expectancy.

## 2. Materials and Methods

### 2.1. Participants

During the measurement period from September to October 2022, participants were recruited by undertaking measurement sessions in the community. Participants were recruited from Niigata City and the suburbs of Shibata City in Niigata Prefecture, Japan. Participants in the study were recruited in writing and orally from the local community. Participants’ criteria were as follows: (1) 65 years of age or older; (2) able to walk independently; (3) living an independent daily life; (4) no paralysis associated with cerebrovascular disease; (5) no numbness or other neurological disease; (6) not training with the intensity of an athlete; (7) no pacemaker inserted; and (8) being Japanese. No sex restrictions were set when recruiting participants. Seventy-two participants took part in the measurement, and three were excluded. The exclusions were because one participant was aged <65 years, and two had measurement errors. The final analysis included 69 participants (72.3 ± 5.7 years, 155 (152–162) cm, and 54.7 (49.9–64) kg). The study was approved by the Ethics Committee of Niigata Bandai Hospital (approval number: 106) and conducted in accordance with the principles of the Declaration of Helsinki. Informed consent was obtained from the participants in writing and orally before the study was conducted.

### 2.2. Study Design and Measurement Items

This study was a cross-sectional observational study that evaluated physical function, body composition, and questionnaires. PhA was calculated for the whole body, including the upper and lower limbs. Motor function was measured using grip strength, F/w as a floor reaction force index when standing up, and the timed up-and-go test (TUG) as an index of walking ability. The Kihon Checklist (KCL) was used to assess frailty, the 25-Geriatric Locomotive Function Scale (GLFS-25) to assess locomotive syndrome, the Life Space Assessment (LSA) to assess the range of physical activity, and the Modified Fall Efficacy Scale (M-FES) to assess fear of falling.

### 2.3. Phase Angle

**Figure 1:**
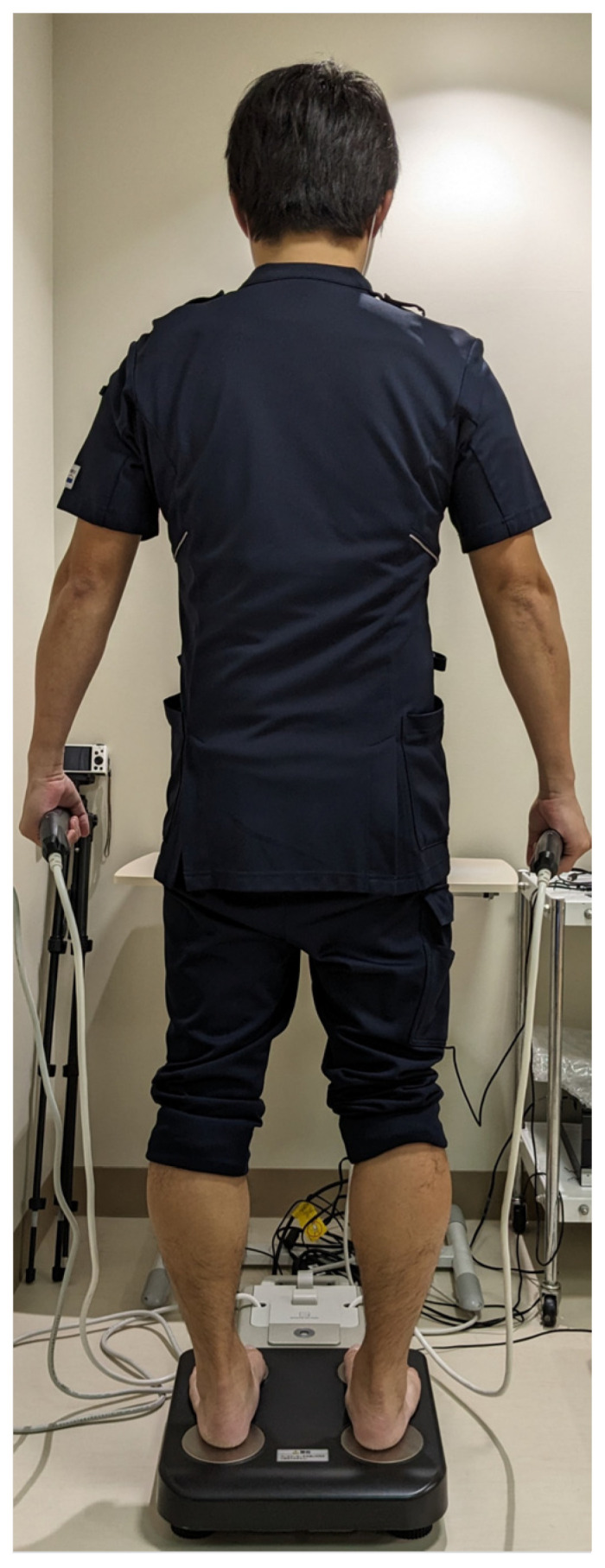
Phase angle measurement using the multi-frequency bioelectrical impedance method.

PhA, as an index of muscle quality, was measured using the BIA method with a multi-frequency eight-electrode body composition analyzer (MC-780A-N, TANITA, Tokyo, Japan). The measuring equipment was the same as that used in a previous study [20], and a weak alternating current of ≤90 μA was applied to the body to measure reactance (Xc) and resistance (R). The measurement frequencies were 50 kHz. During the measurement process, participants were placed in a standing position with bare feet on the toe and heel electrodes, and the hand grip was held with the arm hanging several centimeters away from the body (or waist; Figure 1).

The PhA was calculated using the following formula:

Phase Angle (°) = [arc tangent (Xc/R) × (180/π)]

PhA was calculated as an absolute value. Whole-body PhA was calculated for the left upper limbs, lower limbs, and trunk. The PhAs of the upper and lower limbs were averaged on the left and right sides.

### 2.4. Grip Strength

Grip strength was measured using a grip strength meter (T.K.K5101, TAKEI, Niigata, Japan). The measurement method was the same as in Kobayashi, and the grip dynamometer was held in a standing position [21]. The grip strength of the dominant hand was measured.

### 2.5. TUG

The TUG test is a simple evaluation method that reflects gait and balance functions and was developed by Podsiadlo and Richardson [22]. The participant sits in a chair as the starting posture and upon a signal to start the task, rises from the chair, goes around a pole 3 m away, and the time until he or she is seated again is measured. Participants were instructed to walk quickly and complete the task with maximum effort. The task was performed twice, and the fastest task completion time was taken as the representative value for each participant.

### 2.6. Floor Reaction Force Index during Standing Up

**Figure 2:**
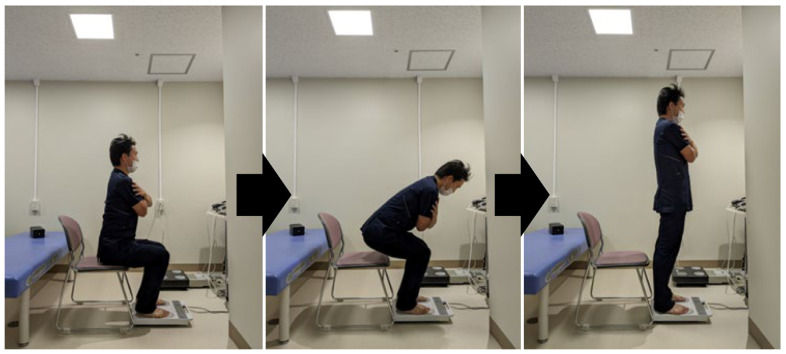
Measurement of the floor reaction force index when standing up.

This index was evaluated using a motor-function measuring device (ZaRitz, TANITA, Tokyo, Japan). The measurements were recorded at a sampling rate of 80 Hz in units of 0.01 kgf/s kg^−1^. The task involved standing up from a seated position in a chair, and the participants stood up three times with maximum effort (Figure 2). Regarding the measurement posture, participants were seated in a chair that was easy to stand up from, and the position of their feet was not specified, but the measurements were taken with their arms crossed in front of their chest. F/w was measured as the coefficient of the ground reaction force obtained during the task operation. f/w (kgf·kg^−1^) is the value obtained by dividing the maximum ground reaction force by body weight and reflects the maximum vertical step force during the chair-rising operation [23]. The rate of change in the maximum increase in ground reaction force among the three trials is shown in the RFD8.75/w as the value obtained by converting the increase in the period of 87.5 ms, including 37.5 ms before and after 12.5 ms when the maximum increase in ground reaction force was recorded, into 1.0 s, and then dividing it by the body weight [23]. F/w is the value obtained when the RFD8.75/w (kgf/s kg^−1^), an index that indicates the rate of change when the maximum increase in ground reaction force is recorded, is the highest [23].

### 2.7. Questionnaire

The KCL was used to evaluate frailty. KCL is a paper questionnaire. The KCL is an index that was developed by the Ministry of Health, Labor and Welfare (MHLW) in Japan at the time of the introduction of a long-term health care prevention project in 2006 and has been used worldwide as the KCL [24], which consists of a 25-item multiple-choice questionnaire, with the severity of frailty determined by the number of items corresponding to each question. The KCL is an evaluation method used to determine the severity of frailty based on the number of responses to each question.

The maximum score is 25 points. Higher scores are interpreted as greater frailty severity compared to lower scores. The cut-off values are as follows: frailty when ≥8 items apply, pre-frail when 4–7 items apply, and robust when ≤3 items apply.

The GLFS-25 was used to evaluate LS and is a 25-item evaluation index created by the Japanese Orthopaedic Association [25]. Although the GLFS-25 is a paper questionnaire, there are items for measuring weight and recording BMI. The degree of LS can be extracted as a continuous variable, and the severity of LS can be evaluated using this score.

The maximum score is 100 points, and the higher the score, the more severe the locomotive syndrome. The cut-off values are as follows. From the total score of GLFS 25, the cut-off value for Robust was ≤6, GLFS 1 was 7 to 16 points, GLFS 2 was 16 to 24 points, and GLFS 3 was 24 points or more.

Fear of falling was assessed using the M-FES [26]—a questionnaire consisting of 14 questions in which participants self-rated their fear of falling on a scale of 0 (not at all confident) to 10 (completely confident), with a maximum score of 140.

Physical activity was evaluated using the LSA [27], which is an evaluation method that calculates a score based on the frequency, range, and degree of independence of each activity during the past month. The minimum score is 0 points, and the maximum score is 120 points; the higher the score, the more active your life is.

### 2.8. Statistical Analysis

The Kolmogorov–Smirnov test was used to check the normality of the data. Normally distributed data are expressed as mean ± standard deviation. Data that were not normally distributed were expressed as the median and interquartile range (interquartile range: IQR). Wilcoxon’s signed-rank test was used to determine the difference between the upper- and lower-limb PhAs. As PhA is an index that is influenced by age, we examined the relationship between PhA at each measurement site and motor function, KCL, and GLFS-25 by using partial correlation coefficients with age as a control variable. The significance level for each statistical analysis was set at *p* < 0.05.

The normality of the variable distribution was verified using the Kolmogorov–Smirnov test, and the significance level was set at *p* < 0.05. No dummy variables were used. Sex was converted into a binary value and analyzed. To confirm linearity in advance, we performed a simple regression analysis of KCL and GLFS-25 with sex, age, upper-limb PhA, lower-limb PhA, and whole-body PhA. The results confirmed linearity between GLFS-25, sex, and lower-limb PhA. There was no linearity between KCL and each independent variable. Therefore, multiple regression analysis was performed using a stepwise method with GLFS-25 as the dependent variable and sex and lower-limb PhA as independent variables.

### 2.9. Ethical Considerations

The study was conducted in accordance with the principles of the Declaration of Helsinki and was approved by the Ethics Committee of Niigata Bandai Hospital (approval number: 106). Participants provided written informed consent to take part in the study.

## 3. Results

### 3.1. Basic Information on Participants and PA

**Figure 3:**
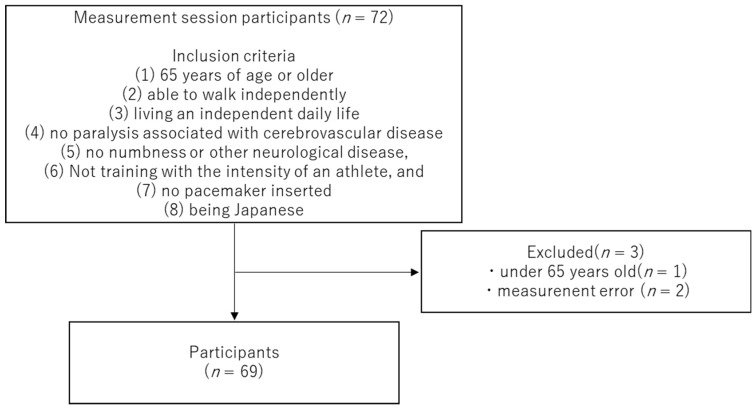
Participants’ screening and selection flowchart.

During the measurement period, 72 individuals were assessed, and 69 participants were included in the analysis after excluding two with body composition data errors and one aged <60 years (Figure 3). Basic information on the participants is shown in Table 1. Normally distributed age, whole-body PhA, lower-limb PhA, and F/w were expressed as mean ± standard deviation. Other data were not normally distributed, so they were expressed as Median (IQR). The participants included 18 males and 51 females. The mean age, height, and weight of the participants were 72.3 ± 5.7 years, 155 (152–162) cm, and 54.7 (49.9–64) kg, respectively (Table 1). The PhA was 4.7 ± 0.5° for the whole body, 5.1 (4.8–5.6°) for the upper limbs and 4.2 ± 0.6° for the lower limbs. Lower-limb PhA was significantly lower than the upper-limb PhA (*p* < 0.05). This result suggests that in these participants, the health of the cells is lower in the lower limbs than in the upper limbs.

### 3.2. Relationship between PhA and Measured Items

Whole-body PhA was significantly correlated with grip strength (r = 0.621, *p* < 0.001), F/w (r = 0.431, *p* < 0.001), and GLFS-25 (r = −0.294, *p* = 0.015). Moreover, upper-limb PhA was significantly correlated with grip strength (r = 0.613, *p* < 0.001) and F/w (r = 0.374, *p* = 0.002). Lower-limb PhA was correlated with grip strength (r = 0.560, *p* < 0.001), TUG (r = −0.251, *p* = 0.039), F/w (r = 0.486, *p* < 0.001), and GLFS-25 (r = −0.365, *p* = 0.002) (Table 2). Lower-limb PhA had a significant correlation with items other than KCL, suggesting that the health of the cells in the lower limbs may be related to motor function and questionnaires that reflect walking and locomotion.

### 3.3. Multiple Regression Analysis

A multiple regression analysis was conducted using a forward stepwise method. The dependent variables were the KCL and GLFS-25 scores, and the independent variables each were PhA, age, and sex.

No independent variables were selected for the multiple regression analysis, with KCL as the dependent variable. Multiple regression analysis with GLFS-25 as the dependent variable selected the lower-limb PhA. The following regression equations were used: The probability level for the significance of the coefficients was *p* < 0.009, the standardized coefficient was −0.314, and the unstandardized coefficient was −3.958. The multiple regression analysis indicated that lower-limb PhA was an independent predictor of the GLFS-25 score (*p* < 0.001) based on the following regression equation:

GLFS-25 = 25.242 + (−3.958 × lower PhA)

The results of the multiple regression analysis suggested that lower-limb PhA is independently related to indicators of locomotive syndrome. It was suggested that the cell health that best reflects the indicators of the locomotive syndrome may be in the lower limbs.

## 4. Discussion

### 4.1. Findings Obtained in This Study

This study was conducted to investigate the differences in the PhA of the upper and lower limbs and their relationship with frailty, LS indices, and motor function in commu-nity-dwelling older adult participants. Lower-limb PhA was significantly lower than up-per-limb PhA in older adults. Furthermore, lower-limb PhA was associated with most items in this study and had significant correlations with all items except KCL. Multiple regression analysis revealed that lower-limb PhA was independently related to the GLFS-25 score. A new feature of this study is that PhA was measured separately in the upper and lower limbs of Japanese older adult participants. Lower-limb PhA was associated with all items except KCL, indicating that the health of lower-limb cells is related to locomotive syndrome and motor functions such as walking and standing.

### 4.2. Basic Information about the Participants and Relationship between PhA and Other Parameters

In this study, the KCL score, an index of frailty, was 3.0 (1–4) points. The KCL cutoff for Japanese individuals is as follows: frailty when ≥8 items apply, pre-frail when 4–7 items apply, and robust when ≤3 items apply [28]. Therefore, it was concluded that the participants in this study were generally robust to pre-frail. The LSA score was 102 (92–120) out of 120, and the M-FES score was 140 (138–140) out of 140. Therefore, the participants performed a sufficient range of activities and had little fear of falling. These results suggest that many participants in this study had mild geriatric diseases and were active throughout their lives. In the present study, the PhA of each measurement site was measured as an index of muscle quality, whereas most of the previous studies on PhA have used the whole-body PhA as an index [17,18]. In a report on 205 Japanese participants with a mean age of 72.6 ± 4.8 years, Uemura et al. [29] found that PhA ranged from 3.9 to 6.7°; however, Homma et al. [20] measured PhA in 19 participants with a mean age of 71.47 ± 4.5 years and reported a PhA of 4.68 ± 0.48°. This value is considered reasonable because it was similar to that obtained in the present study.

Lower-limb PhA was significantly lower than upper-limb PhA, suggesting that PhA differs in each body region. Generally, muscle function decreases with age. Physiological influences are a major factor in muscle function loss, including increased nuclear apoptosis [30], stress from participation [31], muscle fiber denervation [32], reduced satellite cell content and/or regenerative potential [33]. Reduced muscle mass with aging is mainly attributed to smaller type II muscle fiber size [34]. As age-related muscle degeneration occurs earlier in the lower limbs than in the upper limbs [35], we posited that the differences in upper- and lower-limb PhA obtained in this study reflected this age-related change. Furthermore, unlike other PhAs, lower-limb PhA was related to all the assessment items except the KCL score. The motor functions evaluated in this study were grip strength, TUG, and floor reaction force index during standing. Grip strength, which reflects upper-limb muscle function, is possibly related to lower-limb PhA as an index that reflects whole-body muscle strength and correlates with knee extension muscle strength [36]. The KCL and 25-GLFS scores were used to assess frailty and LS, respectively. The KCL score measures frailty but also assesses diverse factors besides mobility, such as social, oral, and cognitive function. Therefore, there was no significant relationship between the KCL score and lower-limb PhA. In contrast, the 25-GLFS comprises many items related to gait, mobility, and physical activity that are significantly related to lower-limb muscle function. Therefore, we considered that lower-limb PhA was related to the 25-GLFS score. Multiple regression analysis showed that the GLFS-25 score was the only factor independently associated with lower-limb PhA. Tanaka et al. reported that systemic PhA was associated with indicators of locomotive syndrome. Whole-body PhA includes lower-limb PhA, but the results of the multiple regression analysis in this study showed that whole-body PhA was not associated with the GLFS-25 score but that lower-limb PhA was found to be independently associated with it. Therefore, the health status of cells in the lower limbs is an effective evaluation indicator for items related to lower-limb function and has been suggested to be related to adult diseases of motor functions, such as walking.

### 4.3. Clinical Applications and Limitations

The results of this study revealed that PhA values differ by the site of measurement and that the related indices differed as well. To the best of our knowledge, this is the first study to classify PhA into upper and lower limbs in a Japanese population and to investigate the relationship with indicators of motor function, frailty, and locomotor syndrome. The clinical application of these findings is that PhA may be associated with various adverse health outcomes [15,16,37]. Furthermore, this study suggests that lower-limb PhA may be an effective evaluation index of activities that are important for independent daily living, such as gait and mobility. The participants of this study had mild degrees of frailty and LS, and lower-limb PhA was associated with gait and standing functions as well as with indicators of geriatric diseases; thus, we infer that lower-limb PhA may be an effective measurement/metric point when promoting independent living. The results of this study indicate that lower-limb PhA is associated with indicators of geriatric disease related to mobility and gait, as well as with getting up and walking. Therefore, the assessment of lower-limb PhA may contribute to the extension of healthy life expectancy.

The key limitations of this study were that: (1) it was difficult to obtain a detailed medical history from a medical perspective, and it was difficult to adjust the measurement time and water intake after meals because the measurements were taken in a local community. Although the participants were recruited based on the condition that they lived independently and could walk alone, it was difficult to ascertain the diseases that they were suffering from. The site or condition of the disease possibly affected the measurements of PhA and motor function. Moreover, the participants had mildly advanced frailty and locomotors—results may be different in individuals with advanced frailty and locomotors. Additionally, since the results of BIA measurements vary depending on the amount of water in the body, detailed investigation that controls food and water intake may be necessary; (2) the PhA is a value that differs between males and females, but the small sample impeded a sex-stratified analysis. Additionally, the small sample size may make it difficult to generalize the results. In the future, it will be necessary to increase the sample size, perform sex-specific analysis, and generalize the data; (3) the study used a cross-sectional design, which precludes the establishment of causal relationships and makes it challenging to infer the direction of the associations between variables; (4) the study was conducted at a single center and included only Japanese older adults, which may limit the generalizability of the findings to other settings or populations; and (5) in this study, grip strength was measured to evaluate upper-limb function and grip strength was related to all items. Therefore, it is considered necessary to add more detailed evaluation indicators for upper-limb function and further consider this with PhA.

## 5. Conclusions

The study’s key finding is that the lower-limb PhA is significantly lower than that of the upper limbs among older Japanese adults in the community. Lower-limb PhA is strongly associated with mobility functions like gait and sit-to-stand performance, as well as indicators of the locomotive syndrome. These results suggest that measuring the lower-limb PhA, instead of the upper-limb or whole-body PhA, may be most relevant for assessing sarcopenia, frailty, and functional status in older populations. The study provides new evidence that PhA assessment should focus on the lower extremities when evaluating age-related muscle quality loss and physical function. The results contribute towards an understanding of the relationship between regional muscle composition and motor decline in aging and can help to guide the development of targeted screening tools and interventions to preserve mobility and independence in older adults. Further research is needed to establish longitudinal changes in limb-specific PhA metrics and their predictive value for geriatric outcomes in diverse populations.

## Figures and Tables

**Table 1:** Basic information of the participants.

Variable	Value
Age, years	72.3 ± 5.7
Height (cm)	155 (152–162)
Weight (kg)	54.7 (49.9–64)
Whole-body phase angle (°)	4.7 ± 0.5
Upper-limb phase angle (°)	5.1 (4.8–5.6)
Lower-limb phase angle (°)	4.2 ± 0.6
Grip strength (kg)	25.9 (23.4–29.2)
Timed Up and Go Test (seconds)	6.1 (5.3–6.5)
F/w (kgf·kg^−1^)	1.3 ± 0.1
Kihon Checklist score (points)	3 (1–4)
25-Geriatric Locomotive Function Scale score (points)	5 (2–12)
Life Space Assessment score (points)	102 (92–120)
Modified Falls Efficacy Scale score (points)	140 (138–140)

Normally distributed data: mean ± standard deviation; Non-normally distributed data: Median value (interquartile range).

**Table 2:** Relationship between the PhA of each measurement site and the measured items.

		Grip Strength	Timed Up and Go Test	F/w	Kihon Checklist	25-Geriatric Locomotive Function Scale Score
Whole-body phase angle	r	0.621	−0.014	0.431	−0.074	−0.294
	*p*	<0.001 *	0.446	<0.001 *	0.549	0.015 *
Upper-limb phase angle	r	0.613	0.010	0.374	−0.027	−0.181
	*p*	<0.001 *	0.938	0.002 *	0.829	0.139
Lower-limb phase angle	r	0.560	−0.251	0.486	−0.134	−0.365
	*p*	<0.001 *	0.039 *	<0.001 *	0.274 *	0.002 *

*: *p* < 0.05.

## Data Availability

The data presented in this study are available on request from the corresponding author. The data are not publicly available due to ethical restrictions.
